# Fluid velocity based simulation of hydraulic fracture—a penny shaped model. Part II: new, accurate semi-analytical benchmarks for an impermeable solid

**DOI:** 10.1007/s11012-018-0903-6

**Published:** 2018-10-24

**Authors:** Daniel Peck, Michal Wrobel, Monika Perkowska, Gennady Mishuris

**Affiliations:** 1 Aberystwyth, UK; 2 AGH University of Science and Technology, Cracow, Poland; 3 EnginSoft S.p.A., Trento, Italy

**Keywords:** Penny-shaped crack, Hydraulic fracture, Universal algorithm, Power law fluid, Leak-off, Numerical solution

## Abstract

In the first part of this paper a universal fluid velocity based algorithm for simulating hydraulic fracture with leak-off was created for a penny-shaped crack. The power-law rheological model of fluid was assumed and the final scheme was capable of tackling both the viscosity and toughness dominated regimes of crack propagation. The obtained solutions were shown to achieve a high level of accuracy. In this paper simple, accurate, semi-analytical approximations of the solution are provided for the zero leak-off case, for a wide range of values of the material toughness and parameters defining the fluid rheology. A comparison with other results available in the literature is undertaken.

**Electronic supplementary material:**

The online version of this article (10.1007/s11012-018-0903-6) contains supplementary material, which is available to authorized users.

## Introduction

Hydraulic fracturing (HF) is the extension of a crack in a solid through application of fluid pressure. It is frequently encountered in both natural (e.g. subglacial drainage) and industrial (e.g. fracking) processes, necessitating a better understanding of the underlying physical phenomena.

Of all the simple 1D models for examining HF, the radial (penny-shaped) formulation is the most important. This is because it is the only one with the potential to accurately portray a three-dimensional system, making it a perfectly suited point of comparison when testing more advanced HF simulators. As a result, having accurate benchmark data for the radial model is of particular importance to the study of hydraulic fracture.

Unfortunately there is not a substantial body of suitable benchmarks available for the radial model. One can mention here the work by Advani et al. [1], where the approximate time-dependent solution for both Newtonian and non-Newtonian fluids is given. However, its accuracy has not been convincingly proved. An early simulator of penny-shaped fracture was presented in [2], where comparison with previous results was also provided. However again, the error level of the final results is unknown. In [3] the asymptotic solutions for zero and large toughness regimes were delivered for a Newtonian fluid. An additional asymptotic solution for the toughness dominated regime, for a Newtonian fluid, over small and large time scales was presented by Bunger et al. [4]. These asymptotic solutions were later shown to correspond reasonably well to experimental results [5].

The field has become more active in the past year however. There is a work of Kanaun [6], which provides a discretized approach to the time-dependent form of the problem. Unfortunately the model only provides an approximate solution for Newtonian fluids in the toughness dominated regime without fluid leak-off. There has also been an experimental paper by Lai et al. [7], which examined the growth of a penny-shaped fracture in a gelatin matrix. This study was able to demonstrate the effect of varying experiment parameters for small values of the fracture toughness, and suggests that such fractures behave according to the scaling arguments of Spence and Sharp [8] over long times. Finally there is a recent numerical solution provided by Linkov [9, 10], for the class of Newtonian and shear-thinning fluids, but only in the viscosity dominated case. Unfortunately, the accuracy of the aforementioned penny-shape benchmarks is still to be confirmed. Additionally, neither of the recalled solutions takes the convenient form of a simple formula (such as those for the KGD model from [11, 12]) that can be easily used for comparison.

In part I of this paper, a numerical algorithm for the simulation of HF, based on the scheme introduced in [11–13], was provided. By employing an appropriate method of fracture front tracing, utilizing the speed equation approach [14], coupled with an extensive use of information on the crack tip asymptotics and regularization of the Tikhonov type (the technical details of both concepts can be found in [15, 16]), it was able to provide high accuracy solutions to the self-similar variant of the penny-shaped model. The relative numerical error of computations was shown to be less than $10^{-7}$, when using $N=300$ nodal points for the spacial mesh. An alternative measure of the computational error, using the known rate of solution convergence, was proposed. It should be noted that this part can be read independently of the original paper, with all relevant information being provided (for a unified version of the text, see arXiv:1612.03307).

The aim of this paper is to utilize the developed high-accuracy algorithm to provide simple solution approximations, which maintain a reasonable level of accuracy, for the zero leak-off case. In addition, the numerical simulations will be used to analyze the accuracy of other benchmarks available in the literature.

The paper is organized as follows. To ensure that both parts of the paper can be read independently, a summary of the results from part I which are needed for this work is provided in Sect. 2, including the definitions and terminology used to describe problem parameters, comprehensive information about the solution asymptotics and a brief overview of the performance of the numerical algorithm. In Sect. 3 numerical reference solutions are given for the variant of an impermeable solid. Simple and accurate solution approximations are delivered for various fixed values of the material toughness, over the whole range of the fluid behaviour index. Next, the computational algorithm is used to verify other solutions available in the literature. Sect. 4 contains the final discussion and conclusions. Some additional information concerning the limiting cases of Newtonian and perfectly plastic fluids, together with respective models of the approximation, is collected in the “Appendix”.

## Self-similar formulation, the speed equation, crack-tip asymptotics and proper variables

In this section we provide a summary of the important relevant results of the first paper. This will include the definition of the self-similar formulation and comments on the function of the algorithm.

### Problem outline and parameters

We examine the problem of a penny-shaped hydraulic fracture. Fluid is pumped in through a source at the fracture opening ($r=0$), with the injection rate being denoted $Q_0$. Because of this, the fracture will grow axisymmetrically about this point and thus modeling through the use of a 1D cross-section is sufficient to describe the problem.

**Table 1:** List of notation

Symbol	Denotes
*w*(*r*, *t*)	Fracture aperture
*l*(*t*)	Fracture length
*p*(*r*, *t*)	Net fluid pressure
*q*(*r*, *t*)	Fluid flow rate
$q_l (r,t)$	Fluid leak-off, assumed smooth and bounded at the crack tip
*v*(*r*, *t*)	Fluid velocity
$Q_0(t)$	Fluid injection (pumping) rate
$K_I(t)$	Stress intensity factor
$K_{Ic}(t)$	Fracture toughness
*E*	Young’s modulus
*M*	Fluid consistency index
*n*	Fluid behaviour index
$\alpha _i$	*ith* exponent of aperture asymptotics
$\beta _i$	*ith* exponent of fluid velocity asymptotics
$\nu$	Poisson ratio
$\Omega (r,t)$	Modified fluid pressure derivative
$\Phi (r,t)$	reduced fluid velocity
$\Psi (t)$	Smooth continuous function defining the self-similar formulation

The fracture’s dimensions will be given by the aperture, *w*(*r*, *t*), and half-length *l*(*t*). We assume that it begins from a pre-existing crack, giving the initial conditions: $w(r,0)=w_*$, $l(0)=l_*$. The net fluid pressure within the fracture, *p*(*r*, *t*), is defined as: $p=p_f-\sigma _0$, where $p_f$ is the total pressure applied to the fracture walls by the fluid and $\sigma _0$ is the confining stress. Fluid leak-off into the surrounding rock, $q_l$, is a predefined smooth function which is bounded at the fracture tip, although no specific formulation is taken during the derivation of the self-similar scheme.

The rheological behaviour of the fluid within the fracture is approximated using a power-law formulation, such that:

1 $$\tau = M {\dot{\epsilon }}^n ,$$

where $\tau$ denotes the shear stress, ${\dot{\epsilon }}$ is the shear strain rate, $0\le n \le 1$ is the fluid behaviour index and *M* is the consistency index. This two-parameter model is too simple to fully incorporate all of the rheological effects associated with hydraulic fracture, however current higher order theories are largely incompatible with the classical representation of penny-shaped HF examined here. Additionally, the power-law formulation is the standard rheological model used when performing analytical examinations of HF. For a fuller description of this, the authors direct the reader to [12, 17].

For simplicity, the following notation is introduced:

2 $$M^\prime = \frac{2^{n+1} (2n+1)^n}{n^n} M , \quad E^\prime = \frac{E}{1-\nu ^2} ,$$

where $M^\prime$ denotes the modified fluid consistency index.

### The speed equation

In order to facilitate the analysis we shall utilize an additional dependent variable, *v*, which describes the average speed of fluid flow through the fracture cross-section [14]. It will be referenced to in the text as the fluid velocity and is defined as:

3 $$v(r,t) = \frac{q(r,t)}{w(r,t)} , \quad v^n (r,t) = -\frac{1}{M^\prime } w^{n+1} \frac{\partial p}{\partial r} .$$

We assume that the leak-off $q_l$ is such that the fluid velocity is finite at the crack tip, meaning that *v* has the following property:

4 $$\lim _{r\rightarrow l(t)} v(r,t) = v_0 (t) <\infty .$$

Additionally, given that the fracture apex coincides with the fluid front (no lag), and that the tip singularity of the leak-off function is weaker than in the Carter law variant, the so-called *speed equation* [18] takes the form:

5 $$\frac{d l}{dt} =v_0(t) .$$

This Stefan-type boundary condition constitutes an explicit method, as opposed to an implicit level set method [19, 20], and can be effectively used to construct an alternative mechanism of fracture front tracing. The advantages of implementing such a condition have been shown in [10–12].

### Self-similar formulation

We define the computational domain in terms of the normalized parameters:

6 $${\tilde{r}} = \frac{r}{l(t)} , \quad {\tilde{t}} = \frac{t}{t_n} , \quad L({\tilde{t}}) = \frac{l(t)}{l_*} , \quad t_n = \frac{M^\prime }{E^\prime } ,$$

such that ${\tilde{r}}\in [0,1]$, while $l_*$ is chosen for convenience.

We introduce the following separation of variables:

7 $$\begin{aligned} w(r,t)&= l_* \Psi ({\tilde{t}}) {\hat{w}}({\tilde{r}}) , \quad&p(r,t) = \frac{M^\prime }{t_n} \frac{\Psi ({\tilde{t}})}{L({\tilde{t}})} {\hat{p}}({\tilde{r}}) , \nonumber \\ q(r,t)&= \frac{l_*^2}{t_n^{\frac{1}{n}}} \frac{\Psi ^{2+\frac{2}{n}} ({\tilde{t}})}{L^{\frac{2}{n}}({\tilde{t}})} {\hat{q}}({\tilde{r}}) , \quad&v(r,t) = \frac{l_*}{t_n^{\frac{1}{n}}} \frac{\Psi ^{1+\frac{2}{n}} ({\tilde{t}})}{L^{\frac{2}{n}}({\tilde{t}})} {\hat{v}}({\tilde{r}}), \nonumber \\ Q_0 (t)&= \frac{l_*^3}{t_n^{\frac{1}{n}}} \frac{\Psi ^{2+\frac{2}{n}} ({\tilde{t}})}{L^{\frac{2}{n}-1}({\tilde{t}})} {\hat{Q}}_0 , \quad&K_{\{I,Ic\}} (t) = E^\prime \sqrt{l_*} \frac{\Psi ({\tilde{t}})}{\sqrt{L({\tilde{t}})}} {\hat{K}}_{\{I,Ic\}} , \end{aligned}$$

$$q_l ({{\tilde{r}}} , {{\tilde{t}}} ) = \frac{l_*}{\gamma t_n^{\frac{1}{n}}} \Psi ^\prime ({{\tilde{t}}} ) {{\hat{q}}}_l ({{\tilde{r}}} ) ,$$

where $\Psi ({\tilde{t}})$ is a smooth continuous function. By separating the variables in this manner it becomes possible to reduce the problem to a time-independent formulation when $\Psi$ is described by an exponential or a power-law type function. From here on the spatial components will be marked by a ’hat’-symbol, and will describe the self-similar quantities. It is worth noting that the separation of spatial and temporal components given in (7) ensures that the qualitative behaviour of the solution tip asymptotics remains the same as in the time-dependent variant.

In the following analysis we take $\Psi$ in the form of a power-law (Table 1):

8 $$\Psi ({{\tilde{t}}} ) = \left( a + {{\tilde{t}}} \right) ^{\gamma } .$$

This form of $\Psi$, alongside the value of *a* and $\gamma$, are taken to ensure consistency with previous examinations in the literature (e.g. [3, 10]). In this case, the fracture length is given by:

9 $$L({\tilde{t}}) = \left[ \left( 1+\frac{2}{n}\right) \rho {\hat{v}}_0\right] ^{\frac{n}{n+2}} \left( a+{\tilde{t}}\right) ^{\gamma +\frac{n}{n+2}} , \quad \rho = \frac{n}{\gamma \left( n+2\right) +n} .$$

#### Crack tip asymptotics

**Viscosity dominated regime** (${\hat{{\boldsymbol{K}}}}_{\boldsymbol{Ic}} = \bf{0}$):

In the viscosity dominated regime the crack tip asymptotics of the aperture and pressure derivative can be expressed as follows:

10 $${{\hat{w}}}({{\tilde{r}}} )= {{\hat{w}}}_0 \left( 1-{\tilde{r}}^2 \right) ^{\alpha _0} + {{\hat{w}}}_1\left( 1-{{\tilde{r}}}^2 \right) ^{\alpha _1}+{{\hat{w}}}_2 \left( 1-{{\tilde{r}}}^2 \right) ^{\alpha _2}+ O\left( \left( 1-{\tilde{r}}^2 \right) ^{\alpha _2 + \delta }\right) , \quad {{\tilde{r}}} \rightarrow 1,$$

11 $$\frac{d{\hat{p}}}{d {\tilde{r}}} ({\tilde{r}})= {\hat{p}}_0 \left( 1-{\tilde{r}}^2\right) ^{\alpha _0-2} + {\hat{p}}_1 \left( 1-{\tilde{r}}^2\right) ^{\alpha _0-1} + O\left( 1 \right) , \quad {\tilde{r}}\rightarrow 1 .$$

The asymptotic behaviour of the pressure function can be derived from the above, however, this form is given due to its use in computations (see the first part of this paper [21] for more details).

As a consequence the fluid velocity behaves as:

12 $${{\hat{v}}} ({{\tilde{r}}} ) = {{\hat{v}}}_0 + {{\hat{v}}}_1 \left( 1-{\tilde{r}}^2 \right) ^{\beta _1} + O\left( \left( 1-{\tilde{r}}^2 \right) ^{\beta _2}\right) , \quad {{\tilde{r}}} \rightarrow 1 .$$

Note that we require ${{\hat{v}}}_0>0$ to ensure the fracture is moving forward. Additionally, it can easily be shown that the following relationship exists between the aperture and fluid velocity tip asymptotics:

13 $${\hat{v}}_0 = \left[ \frac{2n}{(n+2)^2} \cot \left( \frac{n\pi }{n+2}\right) {\hat{w}}_0^{n+2} \right] ^{\frac{1}{n}} .$$

The values of constants $\alpha _i$, $\beta _i$ are given in Table 2. The general formulae for the limiting cases $n=0$ and $n=1$ remain the same as (10)–(12), with the respective powers $\alpha _i$, $\beta _i$ again being determined according to Table 2.

**Table 2:** Values of the basic constants used in the asymptotic expansions for ${\hat{w}}$ and ${\hat{v}}$ for $0<n<1$

Crack propagation regime	$\alpha _0$	$\alpha _1$	$\alpha _2$	$\beta _1$	$\beta _2$
Viscosity dominated	$\dfrac{2}{n+2}$	$\dfrac{n+4}{n+2}$	$\dfrac{2n+6}{n+2}$	1	$\dfrac{2n+2}{n+2}$
Toughness dominated	$\dfrac{1}{2}$	$\dfrac{3-n}{2}$	$\dfrac{5-2n}{2}$	$\dfrac{2-n}{2}$	1

**Toughness dominated regime** (${\tilde{\boldsymbol{K}}}_{\boldsymbol{Ic}}\,\bf{>\,0}$):

Near the fracture front the form of the aperture and fluid velocity asymptotics remains the same as in the viscosity dominated regime (10), (12), however, the multiplier of the aperture leading term can be stated explicitly:

14 $${\hat{w}}_0 = \frac{4}{\sqrt{\pi }}{\hat{K}}_I ,$$

alongside a new relation between the asymptotic terms:

15 $${\hat{v}}_0 = \left[ \frac{(3-n)(1-n)}{4} \tan \left( \frac{n\pi }{2}\right) {\hat{w}}_0^{n+1} {\hat{w}}_1\right] ^{\frac{1}{n}} .$$

Meanwhile, the pressure derivative asymptote yields:

16 $$\frac{d {\hat{p}}}{d {\hat{r}}} ({\tilde{r}}) = {\hat{p}}_0 \left( 1-{\tilde{r}}^2\right) ^{\alpha _1-2} + {\hat{p}}_1 \left( 1-{\tilde{r}}^2\right) ^{\alpha _2-2} + O\left( 1 \right) , \quad {\tilde{r}}\rightarrow 1 .$$

The values of $\alpha _i$, $\beta _i$ for this regime are provided in Table 2. The asymptotics in the limiting cases $n=0$ and $n=1$ is given in “Appendix” (Eqs. (44) and (40) respectively).

#### Behaviour as ${\hat{K}}_{Ic}\rightarrow \infty$

In the previous paper [21], the behaviour of the solution as ${\hat{K}}_{Ic}\rightarrow \infty$ was shown to take the form:

17 $${\hat{w}}({\tilde{r}}) \sim \frac{4}{\sqrt{\pi }}{\hat{K}}_I \sqrt{1-{\tilde{r}}^2} , \quad {\hat{p}}({\tilde{r}}) \sim \frac{\sqrt{\pi }}{2}{\hat{K}}_I , \quad {\hat{v}}_0 \sim \frac{3}{8\sqrt{\pi }{\hat{K}}_I(3-\rho )},$$

18 $${\tilde{r}}{\hat{v}}({\tilde{r}}) = {\hat{v}}_0 \left[ {\tilde{r}}^2 + \frac{3-\rho }{3} \left( 1-{\tilde{r}}^2\right) \right] + O\left( {\hat{K}}_{Ic}^{-1}\right) ,$$

19 $${\tilde{r}}{\hat{q}}({\tilde{r}}) = \frac{\sqrt{1-{\tilde{r}}^2}}{2\pi }\left[ \frac{3{\tilde{r}}^2}{3-\rho } + \left( 1-{\tilde{r}}^2\right) \right] + O\left( {\hat{K}}_{Ic}^{-1}\right) ,$$

where $\rho$, for the case when $\Psi$ is defined by (8), is as stated in (9).

#### The numerical algorithm

The separation of variables used in the self-similar formulation, description of the crack tip asymptotics and limiting behaviour in the case of infinite toughness (given above) provide all of the details we need to define the semi-analytical approximations of numerical solutions and perform comparisons with other benchmarks available in the literature. The full set of governing equations (both the standard and self-similar forms), alongside a complete description of the computational algorithm used to obtain the numerical reference data, are provided in part I of this paper [21] and will not be repeated here.

It should however be stated that the accuracy of the numerical scheme was tested against newly constructed analytical benchmarks and alternative error measures based on the rate of solution convergence. The relative error of the obtained solution is below $10^{-7}$ for all parameters when taking $N=300$ nodal points to define the fracture. The computations converge to the final result in under 20 iterations, obtaining the solution in under 30 seconds when taking $N=300$ boundary nodes. As such, the solution accuracy is more than sufficient to provide a reliable benchmark.

## Numerical results

In this section, the algorithm described in part I of this paper [21] is used to deliver highly accurate numerical benchmark solutions. A comparative analysis with other data available in the literature is given.

### Impermeable solid-reference solutions

With a suitable measure for testing the solution accuracy in place we move onto examining the solution variant most frequently studied in the literature, the case with a zero valued leak-off function and with ${{\hat{Q}}}_0=1$. Although there is no analytical solution to this variant of the problem, due to its relative simplicity, it is commonly used when testing numerical algorithms. For this reason it is very important that credible reference data is provided for this case, which can be easily employed to verify various computational schemes. Both the viscosity and toughness dominated regimes (for different values of the material toughness: ${\hat{K}}_{Ic}=\left\{ 1,10,100\right\}$) will be investigated. In the next subsection, accurate and simple approximations of the obtained numerical solutions will be provided.

#### Semi-analytical benchmark solutions

While the numerical simulator constructed in the first part of the paper [21] is capable of providing high quality reference data, it is not necessarily in a form which can be easily utilized when testing various computational algorithms. Following the idea from [12], we shall also deliver simple and accurate semi-analytical approximations of the numerical solutions, which can easily be used as benchmark examples without the need for advanced computational programs. We provide below formulae mimicking the crack aperture, the fluid velocity and the net fluid pressure.

All the proposed relations preserve the proper asymptotic behaviour at both the fracture origin and tip. They were computed by taking solutions between $n=0.05$ and $n=0.95$, with a step-size of $n=0.05$, and defining approximating functions which predicted each parameter to a desired accuracy. These approximate solution components were then tested against numerical results with a step-size of $n=0.025$, to ensure that the predictions were accurate over the whole range. Respective coefficients (provided in the supplementary material for this paper) used in the approximations have no set length (i.e. the number of significant figures to which they are stated), as the final accuracy of the solution was the deciding factor in their construction.

As a result of this approach each approximated parameter should be treated independently, which means that the guaranteed accuracy does not embrace the mutual interrelations between respective variables (e.g. the fluid velocity computed according to (3) from the approximate ${{\hat{w}}}$ and ${{\hat{p}}}$ is not expected to give the same accuracy as that provided by the approximation for ${{\hat{v}}}$). Moreover, the high level of accuracy of the approximate formulae is guaranteed over the following interval of the fluid behaviour index: $0.05<n<0.95$. The approximations for the limiting cases $n=0$ and $n=1$ are given separately in “Appendix”.

All semi-analytical benchmarks are obtained in the power-law form of the self-similar solution (8), with the following values for the constants:

20 $$a=0 , \quad \gamma = \frac{1}{3}\left( 1-\frac{2n}{n+2}\right) .$$

- **Viscosity dominated regime** (${\hat{{\boldsymbol{K}}}}_{\boldsymbol{Ic}} \,\varvec{=} \,{\bf{0}}$)

For the viscosity dominated regime we propose the following approximations of the dependent variables:

21 $$\begin{aligned} {\hat{w}}_{apx} ({\tilde{r}},n)\,=\, & {} {w}_0 \biggl [ (1-{\tilde{r}}^2)^{\alpha _0}+w_1 (1-{\tilde{r}}^2)^{\alpha _1} +w_2 f_2({\tilde{r}}) +w_3 (1-{\tilde{r}}^2)^{\alpha _1+1}{\tilde{r}}^{2-n} \nonumber \\&+\, w_4 (1-{\tilde{r}}^2)^{\alpha _1+2}{\tilde{r}}^{2-n} + w_5(1-{\tilde{r}}^2)^{5/2}{\tilde{r}}^{3-n} +w_6 f_{1}({\tilde{r}}) \biggr ] , \end{aligned}$$

22 $${\tilde{r}}{\hat{v}}_{apx}({\tilde{r}},n)= v_1 + v_2 (1-{\tilde{r}}^2)+v_3 {\tilde{r}}^{2-n}+v_4(1-{\tilde{r}}^2)^{{\beta _2}} {\tilde{r}}^2 ,$$

23 $$\begin{aligned} {\hat{p}}_{apx}({\tilde{r}},n)= \,& {} {\hat{C}}_p(n) + p_1 {\tilde{r}}^{1-n} + p_2 {\tilde{r}} \left( 1-{\tilde{r}}^2\right) ^{\alpha _0-1} + \frac{p_3}{n} + p_4 {\tilde{r}}\sqrt{1-{\tilde{r}}} \nonumber \\&+ \frac{p_5}{n}\left( 1-{\tilde{r}}\right) ^{\alpha _1-1} + p_6\left( 1-{\tilde{r}}\right) ^{\alpha _1} , \end{aligned}$$

24 $${\hat{v}}_{0,apx} (n)= \sum _{i=0}^7 C_i n^i , \quad {\hat{C}}_p (n) = \frac{\sum _{i=0}^1 D_i n^i}{\sum _{k=0}^3 X_k n^k} ,$$

with:

25 $$f_{1}( {\tilde{r}} )= \sqrt{1-{\tilde{r}}^2}-\frac{2}{3}(1-{\tilde{r}}^2)^{3/2}-{\tilde{r}}^2\log \left| \frac{1+\sqrt{1-{\tilde{r}}^2}}{{\tilde{r}}} \right| ,$$

26 $$f_2({\tilde{r}})= 2 \sqrt{1-{\tilde{r}}^2}+{\tilde{r}}^2 \log \left( \frac{1-\sqrt{1-{\tilde{r}}^2}}{1+\sqrt{1-{\tilde{r}}^2}}\right) .$$

The coefficients $w_i (n)$, $v_i (n)$, $p_i (n)$, $C_i$, $D_i$, $X_k$ are given in the supplementary material, while $\alpha _0$, $\alpha _1$ and $\beta _2$ can be found in Table 2. This formulation is valid for all $0.05< n < 0.95$, with any modifications required in the limiting cases $n =0$ and $n =1$ being outlined in “Appendix”.

Although the self-similar crack propagation speed ${\hat{v}}_0$ can be obtained by evaluating the general formula (22) at the fracture front, an alternative expression (24)$_1$ has been introduced. This is to ensure the highest possible level of accuracy for this important parameter, which is needed both to compute the fracture length $L({\tilde{t}})$, as well as the transformations to alternative schemes from the literature [e.g. (33)].

**Fig. 1:**
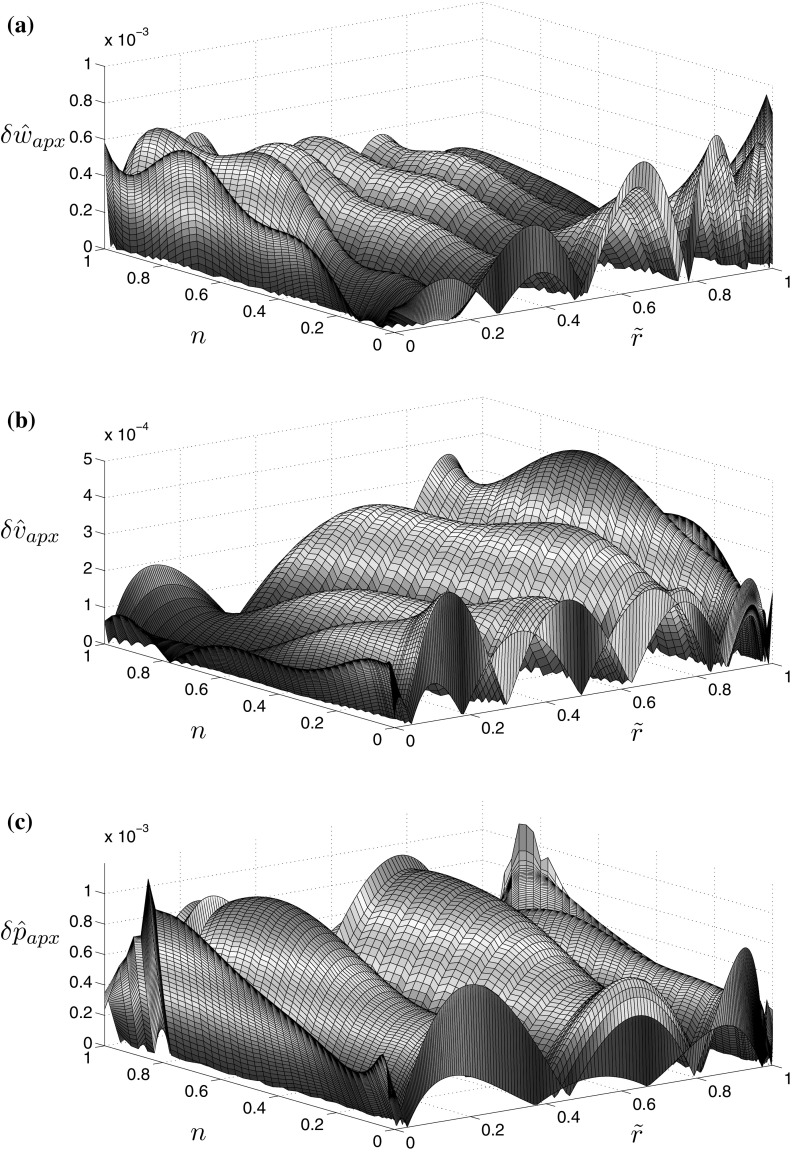
Relative error of the approximations of the numerical solution for **a** the aperture (21), **b** the fluid velocity (22), and the absolute error of approximation of the numerical solution for **c** the fluid pressure (23), in the viscosity dominated regime (${\hat{K}}_{Ic}=0$)

Graphs demonstrating the accuracy of approximations for the aperture, fluid velocity and pressure are provided in Fig. 1. The respective error measures are defined as:

27 $$\delta {\hat{w}}_{apx}({\tilde{r}},n)= \frac{ | {\hat{w}}_n ({\tilde{r}}) - {\hat{w}}_{apx}({\tilde{r}},n)|}{{\hat{w}}_n ({\tilde{r}})} , \quad \delta {\hat{v}}_{apx}({\tilde{r}},n) = \frac{ | {\hat{v}}_n ({\tilde{r}}) - {\hat{v}}_{apx}({\tilde{r}},n) |}{{\hat{v}}_n({\tilde{r}})} ,$$

28 $$\delta {\hat{v}}_{0,apx} ({\tilde{n}})= \frac{| {\hat{v}}_{0,n} - {\hat{v}}_{0,apx}(n) |}{{\hat{v}}_{0,n}} , \quad \delta {\hat{p}}_{apx}({\tilde{r}},n) = | {\hat{p}}_n ({\tilde{r}}) - {\hat{p}}_{apx}({\tilde{r}},n)| ,$$

where ${\hat{w}}_n ({\tilde{r}})$, ${\hat{v}}_n ({\tilde{r}})$, ${\hat{v}}_{0,n}$ and ${\hat{p}}_n ({\tilde{r}})$ are the benchmark solutions obtained by the computational algorithm for a given value of the fluid behaviour index *n*.

**Fig. 2:**
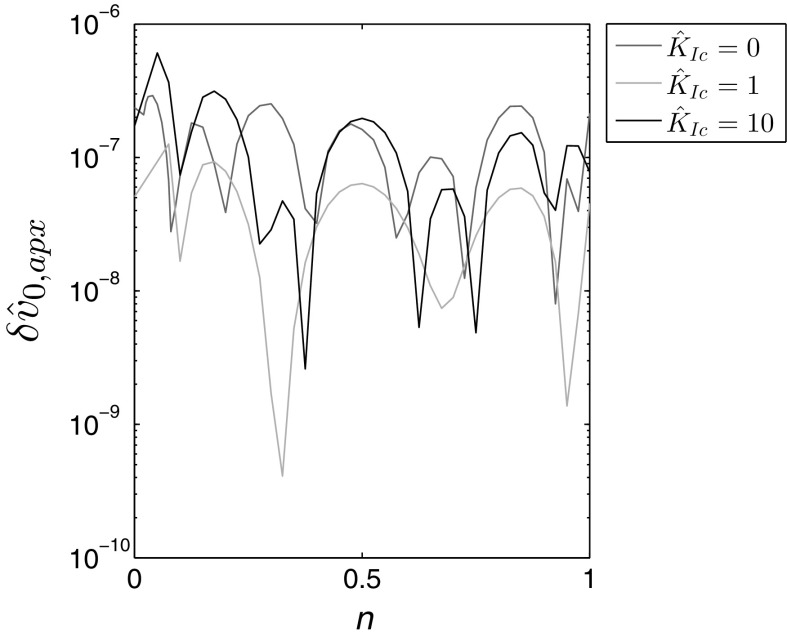
Relative error of approximation for the self-similar crack propagation speed ${\hat{v}}_0$ when evaluated using the specialized equation for ${\hat{v}}_{0,apx}(24)_1$

It can easily be seen that the relative accuracy of the formulae for ${\hat{w}}_{apx}$, ${\hat{v}}_{apx}$, and absolute accuracy for ${\hat{p}}_{apx}$, are of the order $10^{-4}$ over almost the entire interval of *n*. Only for $n=0$ does the error of ${\hat{w}}_{apx}$ slightly exceed $10^{-3}$, while the accuracy of the pressure approximation falls below $10^{-3}$ for specific values of $n>0.8$. The accuracy of ${\hat{v}}_{0,apx}$, computed from (24)$_1$, is reported in Fig. 2. It shows that the relative error is below $2\times 10^{-6}$ for any value of the fluid behaviour index.

- **Toughness dominated regime** (${\hat{{\boldsymbol{K}}}}_{\boldsymbol{Ic}} \,\varvec{=} \,{\bf{0}}$)

In this case the form of the self-similar crack propagation speed approximation, ${\hat{v}}_{0,apx}$, remains as in (24)$_1$. The other solution components are given in the form:

29 $$\begin{aligned} {\hat{w}}_{apx} ({\tilde{r}},n)\,=\, & {} {\hat{w}}_0 \biggl [\sqrt{1-{\tilde{r}}^2}+w_1(1-{\tilde{r}}^2)^{\alpha _1}+w_2 (1-{\tilde{r}}^2)^{3/2}\log (1-{\tilde{r}}^2)\nonumber \\&+\, w_3(1-{\tilde{r}}^2)^{3/2}+ w_4 {\tilde{r}} (1-{\tilde{r}}^2)^{\alpha _2}+w_5 f_{1}({\tilde{r}}) \biggr ] , \end{aligned}$$

30 $${\tilde{r}} {\hat{v}}_{apx} ({\tilde{r}},n)= v_1+v_2(1-{\tilde{r}}^2)^{\beta _1}+v_3 {\tilde{r}}^{2-n}+v_4(1-{\tilde{r}}^2) ,$$

31 $${\hat{p}}_{apx}({\tilde{r}},n)= p_1+p_2 f_3 ({\tilde{r}},n)+ p_3 (1-{\tilde{r}}^2)^{\alpha _1-1}+p_4 {\tilde{r}}^{1-n} ,$$

with:

32 $$f_3 ({\tilde{r}},n) =\alpha _1 \sqrt{\pi } \frac{\Gamma (\alpha _1)}{\Gamma (\alpha _1+1/2)} {_2}F_1 \left( 1, \frac{n-2}{2},\frac{1}{2}, r^2\right) ,$$

where ${\hat{w}}_0$ is given by (14), $f_1$ takes the form (25), and $\alpha _1$ is in Table 2. The coefficients $w_i (n)$, $v_i (n)$, $p_i(n)$, $C_i$ are given in the supplementary material for ${\hat{K}}_I=\left\{ 1 , 10 \right\}$. For $n=\left\{ 0,1\right\}$ some parameters require alternate representations, which are outlined in “Appendix”.

**Fig. 3:**
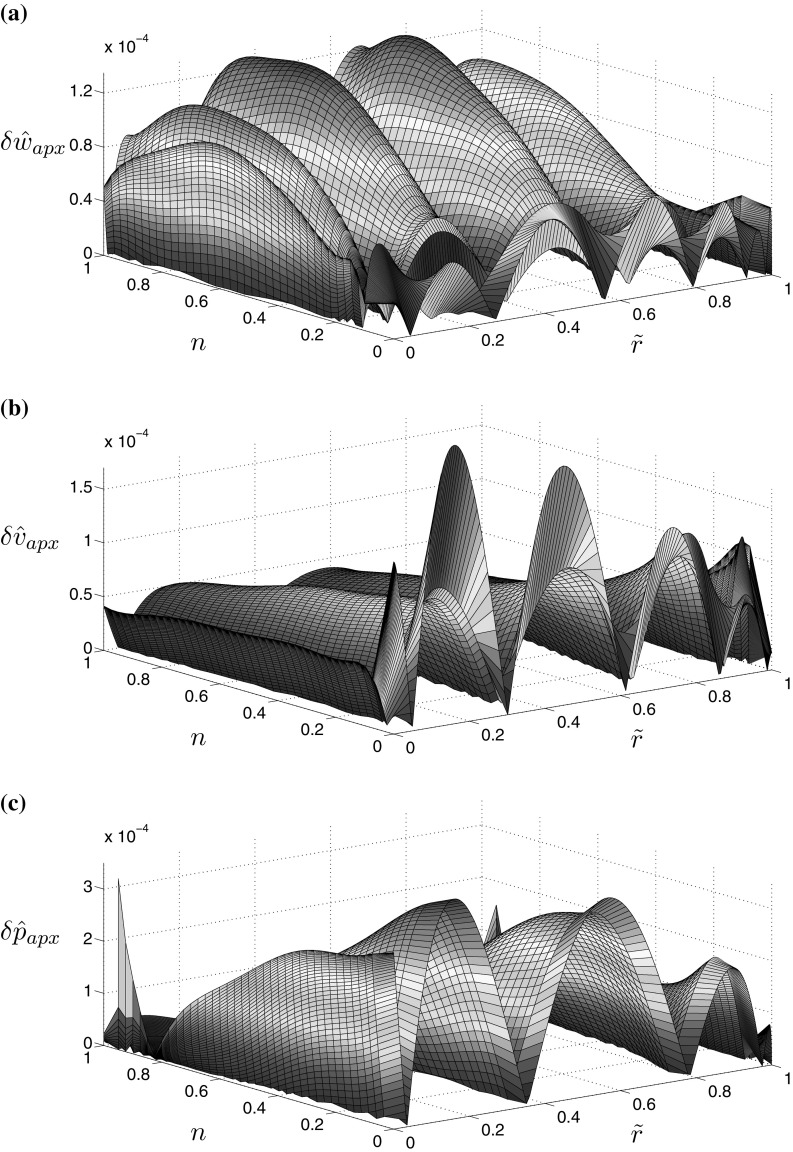
Relative error of the approximations of the numerical solution for **a** the aperture (29), **b** the fluid velocity (30), and the absolute error of approximation of the numerical solution for **c** the fluid pressure (31), in the toughness dominated regime with ${\hat{K}}_I=1$

**Fig. 4:**
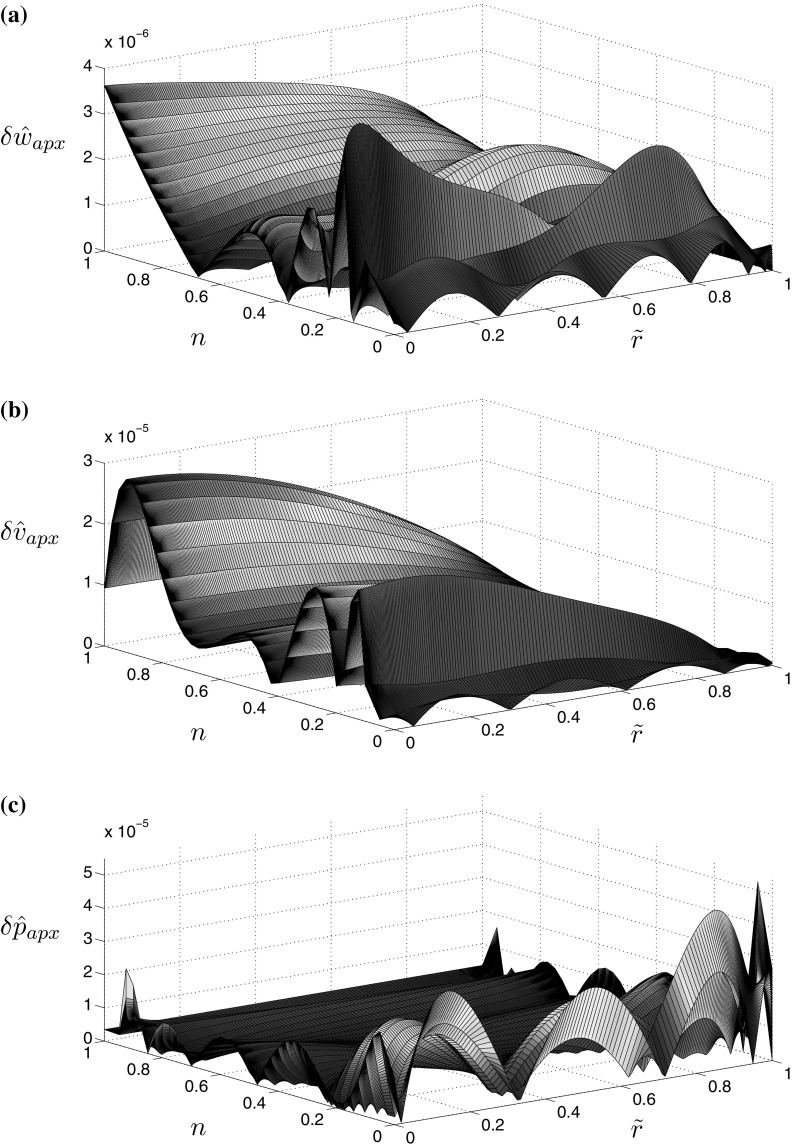
Relative error of the approximations of the numerical solution for **a** the aperture (29), **b** the fluid velocity (30), and the absolute error of approximation of the numerical solution for **c** the fluid pressure (31), in the toughness dominated regime with ${\hat{K}}_I=10$

This time the quality of approximations is better than those for the viscosity dominated regime (see Figs. 3, 4). For ${\hat{K}}_{Ic}=1$ the approximation errors do not exceed $3\times 10^{-4}$, regardless of the considered variable or the value of the fluid behaviour index *n*. When analyzing the case ${\hat{K}}_{Ic}=10$ one can see that the accuracy of approximations improved even further, being up to two orders of magnitude better than that for ${\hat{K}}_{Ic}=1$.

#### Verification of other results from the literature

In the following, using our highly accurate numerical scheme, we will verify the results provided so far by other authors. Unfortunately, there are only a handful of papers where respective data is provided in a form which enables comparison. In most cases only graphs of the dependent variables are given. In order to make sure that respective results are comparable, the zero leak-off case will again be examined, taking fixed ${\hat{Q}}_0=1$, with transformations between the schemes outlined as necessary. Throughout this section we will use $N=300$ nodal points, which in previous the first paper (Part I) [21] we have shown is accurate to 7 significant digits.

We begin by analyzing the solution delivered by Linkov in [10] for the viscosity dominated regime (${\hat{K}}_{Ic}=0$). Note that, as slightly different normalizations are used to obtain the self-similar solution, the following transformations are required to obtain a comparison between the results:

33 $$\begin{aligned} {\hat{w}} ({\tilde{r}})=\, & {} \zeta ^{\frac{n}{n+2}} {\hat{w}}^L ({\tilde{r}} ), \quad {\hat{p}} ({\tilde{r}}) = \zeta ^{\frac{n}{n+2}} {\hat{p}}^L ({\tilde{r}} ) , \nonumber \\ {\hat{v}} ({\tilde{r}})=\, & {} \zeta {\hat{v}}^L ({\tilde{r}} ) , \quad {\hat{Q}}_0= \frac{1}{\xi _{*,n}^3} \zeta ^{\frac{2\left( n^2+2\right) }{n+2}} {\hat{Q}}_0^L , \nonumber \\ {\hat{q}}_l ({\tilde{r}})=\, & {} \zeta ^{\frac{n}{n+2}} {\hat{q}}_l^L ({\tilde{r}} ) , \quad \xi _{*,n}= \left( 2\pi \int _0^1 \varsigma {\hat{w}}^L (\varsigma ) \, d\varsigma \right) ^{-\frac{1}{3}} , \end{aligned}$$

where

34 $$\zeta = \frac{3{\hat{v}}_0\left( n+2\right) }{2n+2} .$$

Here $\xi _{*,n}$ is Linkov’s normalized fracture length when $Q_0=1$. It can easily be shown using the equation for fracture length (9) that, in order for the two formulations to coincide, the following scaling condition must be met:

35 $$\xi _{*,n}= \zeta ^{\frac{2\left( n+1\right) }{3\left( n+2\right) }} .$$

The values of the self-similar fracture opening, crack propagation speed and fracture half-length are shown in Table 3. The results obtained in [10] are included for completeness, and denoted with a superscript *L*. The notation ${\hat{w}}^T (0)$ represents the transformed crack opening computed according to (33)$_1$ [this value is to be compared with ${\hat{w}}^L (0)$].

**Table 3:** The values of fracture opening, crack propagation speed and half-length, given to an accuracy of seven significant figures (which defines the solution accuracy achievable for $N=300$ using the authors’ solver)

n	${\hat{v}}_0$	${\hat{w}}(0)$	${\hat{w}}^T (0)$	$\xi _{*,n}$	${\hat{w}}^L (0)$	$\xi _{*,n}^L$
0	0.1314342	1.688787	1.688787	0.7332914	1.6889	0.7330
0.1	0.1427914	1.602559	1.672277	0.7317711	1.6724	0.7318
0.2	0.1527660	1.535686	1.661661	0.7295243	1.6617	0.7296
0.3	0.1615208	1.482567	1.655773	0.7267291		
0.4	0.1691971	1.439637	1.653833	0.7235073	1.6537	0.7236
0.5	0.1759138	1.404539	1.655334	0.7199395		
0.6	0.1817680	1.375680	1.659981	0.7160755	1.6599	0.7162
0.7	0.1868366	1.351968	1.667648	0.7119399		
0.8	0.1911776	1.332662	1.678369	0.7075363	1.6784	0.7076
0.9	0.1948308	1.317280	1.692338	0.7028480		
1	0.1978175	1.305555	1.709934	0.6978375	1.7092	0.6978

The final two columns, denoted with superscript *L*, show the values provided in [10]. The symbols ${\hat{w}}^T$ and $\xi _{*,n}$ stand for the transformed fracture opening and fracture half-length computed according to (33). These values are to be compared with the last two columns

It can easily be seen that there is a high level of correspondence between the results in this paper and those provided by Linkov for different values of the fluid behaviour index *n*. The maximum relative discrepancy is of the order $4.3\times 10^{-4}$, which considering the accuracy of our solution demonstrated in the previous paper, describes the level of accuracy achieved by the solution from [10]. We note that, in our approach, it is sufficient to take merely $N=40$ points to have a similar accuracy.

Another solution to be analyzed is that from Savitski and Detournay [3], which provides asymptotic approximations for both the viscosity and toughness dominated regimes in the case of a Newtonian fracturing fluid. The interrelations between the self-similar crack opening and crack propagation speed given in [3] and our results are as follows:

36 $${\bar{\Omega }}_{m,0}({\tilde{r}}) = \left[ \frac{4}{9{\hat{v}}_0}\right] ^{\frac{1}{3}} {\hat{w}}({\tilde{r}}) , \quad V({\tilde{r}})=\frac{4}{9 {\hat{v}}_0} {\hat{v}}({\tilde{r}}) .$$

Savitski and Detournay specify the following asymptotic approximation for the self-similar aperture:

37 $${\bar{\Omega }}_{m,0} ({\tilde{r}}) = 2^{\frac{1}{3}} \times 3^{\frac{1}{6}} \left( 1-{\tilde{r}}^2 \right) ^{\frac{2}{3}} + O\left( \left( 1-{\tilde{r}}^2 \right) ^{\frac{5}{3}} \right) ,\quad {\tilde{r}}\rightarrow 1 .$$

Using the relevant transformations yields:

38 $${\hat{w}}({\tilde{r}}) = 2^{\frac{1}{3}} \times 3^{\frac{1}{6}} \left[ \frac{9{\hat{v}}_0}{4}\right] ^{\frac{1}{3}} \left( 1-{\tilde{r}}^2 \right) ^{\frac{2}{3}} + O\left( \left( 1-{\tilde{r}}^2 \right) ^{\frac{5}{3}} \right) , \quad {\tilde{r}}\rightarrow 1.$$

Note that interrelation between ${{\hat{w}}}_0$ and ${{\hat{v}}}_0$ resulting from (38) is exactly the same as the one given by (13) based on the speed equation. Thus, any solution in the viscosity dominated regime (for $n=1$) preserving the latter will be equivalent in terms of ${{\hat{w}}}_0$ and ${{\hat{v}}}_0$ to the data provided in [3].

For the toughness dominated regime it is unfortunately not possible to perform the same comparison as above with the results from [3]. This is due to the fact that Savitski and Detournay’s solution is only self-similar in the limiting cases $K_I=\left\{ 0,\infty \right\}$, and is a time dependent function of $K_I (t)$ in the interim.

**Fig. 5:**
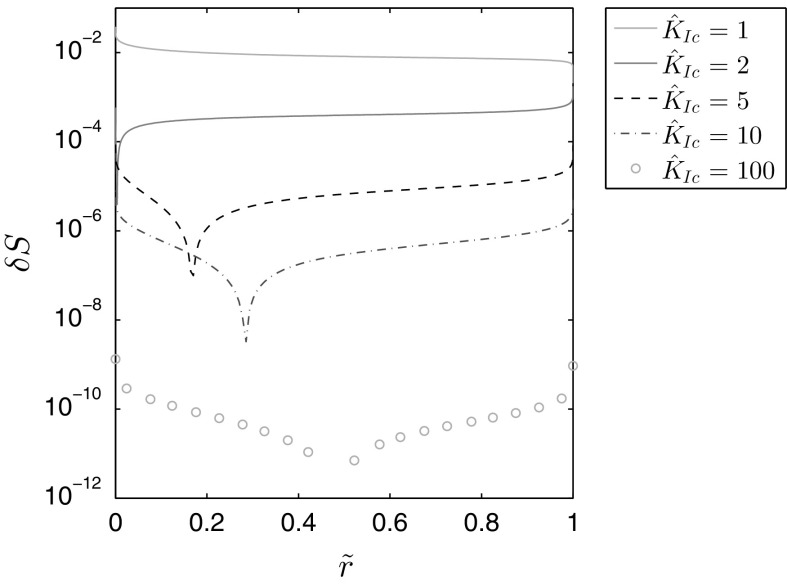
Comparison of the ratio between the fracture aperture and pressure for Savitski and Detournay’s solution [3] and that presented in this paper for a few values of the fracture toughness. Here $\delta S$ shows the relative error

It is however possible to check the ratio between the fracture pressure and aperture with the following equality:

39 $$\frac{{\hat{w}}({\tilde{r}})}{{\hat{p}}({\tilde{r}})} = \frac{\Omega _{k}({\tilde{r}})}{\gamma _0 \Pi _{k} ({\tilde{r}})} ,$$

where $\Omega _k$ is Savitski and Detournay’s normalized aperture, $\Pi _k$ is the normalized pressure and $\gamma _0=\left( 3/\pi \sqrt{2}\right) ^{\frac{2}{5}}$ is the first term of the normalized asymptotic expansion of the fracture length [3]. Noting that the paper gives the limiting values for $K_{Ic}\rightarrow \infty$ as being $\Omega _{k,0}=\left( 3/8\pi \right) ^{\frac{1}{5}}\sqrt{1-{\tilde{r}}^2}$ and $\Pi _{k,0}=\pi \left( \pi / 12 \right) ^{\frac{1}{5}} / 8$, one can easily determine from (17) that ratio (39) is satisfied in the limit. As such, we can evaluate the validity of the asymptotic fromulae from [3] by examining the relative ratio between the two sides of (39), which we will label $\delta S$. The results for this metric, pertaining to the values ${\hat{K}}_I=\left\{ 1,2,5,10,100\right\}$, are provided in Fig. 5.

It is evident from this comparison that there is a clear correspondence between the results of this paper and those obtained by Savitski and Detournay. The disparity between respective data in the large toughness case, ${\hat{K}}_{Ic}=100$, is compatible with the error of our solution demonstrated for this model in the previous paper. This is a strong verification of the validity of the asymptotic formulae from [3]. However, the accuracy of those approximations diminishes greatly for lower values of the fracture toughness, with an error of order $10^{-1}$ when ${\hat{K}}_I=1$. This, in turn, provides us with an estimate of when the formula in [3] loses its practical applicability.

## Conclusions

In this paper, highly accurate numerical reference solutions for a penny-shaped hydraulic fracture in the case of an impermeable solid have been delivered. Simple and accurate approximate formulae mimicking these solutions, over whole range of the fluid behaviour index, have been given for fixed values of the material toughness. These constitute a set of accurate and easily accessible reference solutions when investigating the performance of other computational algorithms. Verification of other results available in the literature has been performed.

### Electronic supplementary material

Below is the link to the electronic supplementary material.

Supplementary material 1 (pdf 149 KB)

## Appendix: Asymptotics and semi-analytical approximations for the limiting cases: Newtonian and plastic fluids

### Newtonian fluid: $n=1$

#### Basic formulae

In the case of a Newtonian fluid the majority of the results remains the same as in the general case (setting $n=1$), but a few constants and functions will take alternate forms. These are detailed below.

The crack tip asymptotics in the viscosity dominated regime can be described by general relations (10)–(12). However, in the toughness dominated mode one has:

40 $$\begin{aligned} {{\hat{w}}}({{\tilde{r}}})\,=\, & {} {{\hat{w}}}_0 \sqrt{1-{\tilde{r}}^2 } + {{\hat{w}}}_1 \left( 1-{{\tilde{r}}}^2 \right) + {{\hat{w}}}_2\left( 1-{{\tilde{r}}}^2 \right) ^{\frac{3}{2}}\log \left( 1-{\tilde{r}}^2 \right) \nonumber \\&+\, O\left( \left( 1-{\tilde{r}}^2 \right) ^{\frac{3}{2}}\right) , \quad {{\tilde{r}}} \rightarrow 1, \end{aligned}$$

41 $$\frac{d {\hat{p}}}{d {\tilde{r}}}= {\hat{p}}_0 \left( 1-{\tilde{r}}^2\right) ^{-1} + {\hat{p}}_1 \left( 1-{\tilde{r}}^2\right) ^{-\frac{1}{2}}+ O\left( 1 \right) , \quad {\tilde{r}}\rightarrow 1 .$$

#### Semi-analytical approximation

The semi-analytical approximations for the aperture and fluid velocity remain the same as those presented in Sect. 3.1.1, however, the form of the pressure function must be modified. We now have:

- The viscosity dominated regime ($K_{Ic}={\text0}$):

Here the form of the aperture approximation (21) remains the same as in the general case, but the approximations of the fluid velocity and pressure now become:

42 $$\begin{aligned} {\hat{p}}_{apx}({\tilde{r}},n)&= {\hat{C}}_p(n) + p_1 \log ({\tilde{r}}) + p_2 {\tilde{r}}\left( 1-{\tilde{r}}^2\right) ^{-\frac{1}{3}} + p_3 + p_4 {\tilde{r}}\sqrt{1-{\tilde{r}}} \nonumber \\&+\, p_5\left( 1-{\tilde{r}}\right) ^{\frac{2}{3}} + p_6\left( 1-{\tilde{r}}\right) ^{\frac{5}{3}}, \end{aligned}$$

with ${\hat{C}}_p(n)$ defined by (24)$_2$.

- The toughness dominated regime ($K_{Ic}>{\text 0}$):

The form of the aperture (29) and fluid velocity (30) approximations are the same as in the general case, but the approximation of the pressure is now:

43 $${\hat{p}}_{apx}({\tilde{r}},n) = p_1+p_2 \log (1-{\tilde{r}}^2)+p_3\log ({\tilde{r}}) +p_4 {\tilde{r}} \sqrt{1-{\tilde{r}}} .$$

### Perfectly plastic fluid: $n=0$

#### Basic formulae

The crack tip asymptotics in the viscosity dominated regime remains in the same form as was outlined in (10)–(12). In the toughness dominated mode however it now yields:

44 $$\begin{aligned} {{\hat{w}}}({{\tilde{r}}})& = {{\hat{w}}}_0 \sqrt{1-{\tilde{r}}^2 }+ {{\hat{w}}}_1\left( 1-{{\tilde{r}}}^2 \right) ^{\frac{3}{2}}\log \left( 1-{\tilde{r}}^2 \right) + {{\hat{w}}}_2\left( 1-{{\tilde{r}}}^2 \right) ^{\frac{3}{2}} \nonumber \\&+ O\left( \left( 1-{\tilde{r}}^2 \right) ^{\frac{5}{2}}\right) , \quad {{\tilde{r}}} \rightarrow 1, \end{aligned}$$

45 $$\frac{d {\hat{p}}}{d {\tilde{r}}}= {\hat{p}}_0 \left( 1-{\tilde{r}}^2\right) ^{-\frac{1}{2}} + O\left( 1 \right) , \quad {\tilde{r}}\rightarrow 1 .$$

#### Semi-analytical approximation

As a result of these changes to the system behaviour and asymptotics, the semi-analytical approximations presented in Sect. 3.1.1 take the following form when $n=0$:

- The viscosity dominated regime ($K_{Ic}=0$):

Here the form of the aperture approximation (21) remains the same as in the general case, however, the approximations of the fluid velocity and pressure are now:

46 $${\tilde{r}}{\hat{v}}_{apx} ({\tilde{r}},n)= \left( v_1 {\tilde{r}} + v_2 \right) / \left( {\tilde{r}}^3 =+ v_3 {\tilde{r}}^2 + v_4 {\tilde{r}} + v_5 \right) ,$$

47 $$\begin{aligned} {\hat{p}}_{apx}({\tilde{r}},n)& = {\hat{C}}_p(n) + p_1 {\tilde{r}} + p_2 {\tilde{r}}\log \left( 1-{\tilde{r}}\right) + p_3 + p_4 {\tilde{r}}\sqrt{1-{\tilde{r}}} \nonumber \\&+\, p_5\left( 1-{\tilde{r}}^2\right) \log \left( 1-{\tilde{r}}^2\right) + p_6\left( 1-{\tilde{r}}\right) + p_7 \left( 1-{\tilde{r}}\right) ^2 , \end{aligned}$$

with ${\hat{C}}_p(n)$ defined by (24)$_2$.

- The toughness dominated regime ($K_{Ic}>0$):

The pressure approximation (31) is the same as in the general case, however, the aperture and fluid velocity approximations become:

48 $$\begin{aligned} {\hat{w}}_{apx}({\tilde{r}},n)& = {\hat{w}}_0 (\sqrt{1-{\tilde{r}}^2}+w_1(1-{\tilde{r}}^2)^{3/2}+w_2 (1-{\tilde{r}}^2)^{3/2}\log (1-{\tilde{r}}^2)\nonumber \\&+\, w_3 (1-{\tilde{r}}^2)^3\log (1-{\tilde{r}}^2)+ w_4 (1-{\tilde{r}}^2)^{5/2}{\tilde{r}}^2+w_5 f_{\Omega }) , \end{aligned}$$

49 $${\tilde{r}}{\hat{v}}_{apx}({\tilde{r}},n)= v_1+v_2(1-{\tilde{r}}^2)^2 \log (1-{\tilde{r}}^2)+v_3 (1-{\tilde{r}}^2)^2+v_4(1-{\tilde{r}}^2)^2 {\tilde{r}}^2 \log ({\tilde{r}}) ,$$

with $f_\Omega$ being given in (25) and ${\hat{w}}_0$ in (14).
