# Supplementary material for: Fluid velocity based simulation of hydraulic fracture—a penny shaped model. Part II: new, accurate semi-analytical benchmarks for an impermeable solid
Source: Meccanica. 2018 Oct 24;53(15):3637–50. doi: 10.1007/s11012-018-0903-6 (PMC6405074; doi:10.1007/s11012-018-0903-6)
Supplement: Supplementary file 1 — Supplementary material 1 (pdf 149 KB) [file 11012_2018_903_MOESM1_ESM.pdf]

Fluid velocity based simulation of hydraulic fracture - a penny shaped model. Part II: New, accurate semi-analytical benchmarks for an impermeable solid
Supplementary material

D. Peck, M. Wrobel, M. Perkowska, G. Mishuris

1 Coefficients of the approximate solutions

For any value of the fluid behaviour index n and self-similar material toughness $\hat{K}_I$, the self-similar crack propagation speed $\hat{v}_0$ is given in the form $(24)_1$. The values of respective coefficients C_i are provided in Table S1 for $\hat{K}_I = \{0, 1, 10\}$.

$\hat{K}_I$	C_0	C_1	C_2	C_3
0	0.1314342	0.1210766	-0.0781383	0.031537
1	0.06125898	0.050859704	-0.029318586	0.012385442
10	7.04065×10^{-3}	3.602954×10^{-3}	-2.00895×10^{-3}	1.373533×10^{-3}

$\hat{K}_I$	C_4	C_5	C_6	C_7
0	-5.293135×10^{-3}	-6.62796×10^{-3}	5.350374×10^{-3}	-1.521311×10^{-3}
1	-2.920989×10^{-3}	-2.8172727×10^{-4}	4.8397784×10^{-4}	-1.2631848×10^{-4}
10	-1.0841455×10^{-3}	7.441777×10^{-4}	-3.330152×10^{-4}	6.79385×10^{-5}

Table S1: Values of the coefficients C_i used to approximate $\hat{v}_0$ (24) for different values of the fracture toughness.

Meanwhile, the coefficients of the constant $\hat{C}_p(n)$, which takes the form $(24)_2$ in the viscosity dominated case ($\hat{K}_{Ic} = 0$), are provided in Table S2.

The remaining coefficients for the fracture aperture, pressure and fluid velocity approximations are outlined for different values of the fracture toughness below.

D_0	D_1	X_0	X_1	X_2	X_3
3.5484	-3.1946	3.711	-1.3516	-3.3625	1

Table S2: Values of the coefficients D_i, X_k used to approximate the constant $\hat{C}_p(n)$ in equation $(24)_2$.

1.1 Viscosity dominated regime ($\hat{K}_{Ic} = 0$)

In the general case $0 < n < 1$ the coefficients of approximation for the aperture (21), fluid velocity (22) and pressure (23) are given as:

$$\mathcal{Z}(n) = \frac{\sum_{k=0}^{5} r_k n^k}{(1-n)^\kappa \sum_{k=0}^{5} s_k n^k}, \tag{S1}$$

with the values of r_k, s_k and κ for the case $0 < n < 1$ being listed in Tables S3 and S4.

In the case of a Newtonian fluid $n = 1$ the coefficients used to approximate the aperture (21) and the fluid velocity (22) remain the same as in the general case. The coefficients of the pressure approximation (42) are now given by:

$$p_1 = -0.0715, \quad p_2 = -0.22233, \quad p_3 = 114.7455, \tag{S2}$$
$$p_4 = 0.0413, \quad p_5 = -0.12312, \quad p_6 = -0.0237.$$

For the perfectly plastic fluid $n = 0$ the coefficients used to approximate the aperture (21), fluid velocity (46) and pressure (47) are as follows:

$$w_0 = 1.773, \quad w_1 = 0.06, \quad w_2 = -0.1638, \quad w_3 = 0.335,$$
$$w_4 = -0.289, \quad w_5 = -0.179, \quad w_6 = 0.6607,$$
$$v_1 = 4.8656, \quad v_2 = 1.703, \quad v_3 = -20.484, \quad v_4 = 51.4, \quad v_5 = 18.07, \tag{S3}$$
$$p_1 = -0.5921, \quad p_2 = 0.28201, \quad p_3 = 0.264, \quad p_4 = 0.127,$$
$$p_5 = 0.099, \quad p_6 = -0.6436, \quad p_7 = 0.3806.$$

1.2 Toughness dominated regime with $\hat{K}_I = 1$

In the general case $0 < n < 1$ the approximation coefficients for the aperture (29), fluid velocity (30) and pressure (31) are given in the form:

$$\mathcal{Z}(n) = \frac{\sum_{k=0}^{7} r_k n^k}{\sum_{k=0}^{5} s_k n^k}, \tag{S4}$$

with the values of r_k, s_k for the case $0 < n < 1$ being listed in Tables S5 and S6.

For the Newtonian fluid $n = 1$ the coefficients for the approximate pressure function (43) are now given by:

$$p_1 = 0.90064, \quad p_2 = 9.053 \times 10^{-3}, \quad p_3 = -0.0126243, \quad p_4 = -7.3525 \times 10^{-4}, \tag{S5}$$

while those for the aperture (29) and fluid velocity (30) remain the same as in the general case.

$\mathcal{Z}(n)$	r_0	r_1	r_2	r_3	r_4	r_5
w_0	1.087913	0.629465	0.1884191	-0.0954601	0.0539965	0
w_1	0.0731578	-0.0940037	-0.2924713	0.712854	-0.220774	0
w_2	-0.0813068	0.1374238	-0.0672009	-0.0557795	0	0
w_3	0.1130671	-0.458432	-0.549883	0	0	0
w_4	-0.3394015	1.968425	-0.324536	0	0	0
w_5	-0.4207775	2.729404	0	0	0	0
w_6	0.374811	-0.595337	0.4492	0.0240865	0	0
v_1	- 0.0618879	0.238355	0.488614	-0.089777	0	0
v_2	0.106085	-0.0105322	-0.43386	-0.0150819	0	0
v_3	0.0260021	0.0203881	-0.0379568	0.0258418	-6.69655×10^{-3}	0
v_4	-0.0127769	-0.0152235	0.0201527	0	0	0
p_1	-1.383	0.6689	0	0	0	0
p_2	-18.738	-7.314	7.802	0	0	0
p_3	9.470147	-26.2166	23.92346	-7.16925	0	0
p_4	0.1491	-0.09304	-0.13218	0.16745	-0.12976	0.07958
p_5	-0.0754673	-0.463258	1.755936	-1.882529	0.732565	-0.05901
p_6	-27.292	-94.974	111.858	0	0	0

Table S3: The values of coefficients r_i used in approximation (S1) in the general case $0 < n < 1$ with $\hat{K}_I = 0$.

$\mathcal{Z}(n)$	s_0	s_1	s_2	s_3	s_4	s_5	κ
w_0	0.613792	1	0	0	0	0	0
w_1	1.30785	-1.57716	0.820255	1	0	0	0
w_2	0.504215	-0.2551376	-0.436244	1	0	0	0
w_3	0.2952694	0.319092	-0.2805504	0.0738782	1	0	0
w_4	1.022663	0.28412	-1.162825	1.77880	1	0	0
w_5	2.02325	-0.427459	-1.46776	3.51378	1	0	0
w_6	0.57009	-1.09863	1	0	0	0	0
v_1	-3.3351×10^{-6}	2.37989	1	0	0	0	0
v_2	-5.84509×10^{-6}	4.0795	2.2347	1	0	0	0
v_3	-2.50863×10^{-6}	1	0	0	0	0	0
v_4	1.75635	0.685504	1	0	0	0	0
p_1	2.3357	4.248	2.4022	1	0	0	1
p_2	0	33.212	47.87	1	0	0	0
p_3	16.78564	-30.8988	8.118	9.44912	-4.39755	1	0
p_4	1	0	0	0	0	0	0
p_5	0.133757	0.959086	-2.15943	1	0	0	0
p_6	495.12	686.2	-734.6	1	0	0	0

Table S4: The values of coefficients s_i and κ used in approximation (S1) in the general case $0 < n < 1$ with $\hat{K}_I = 0$.

In the case of a perfectly plastic fluid $n = 0$ the coefficients used to approximate the aperture (48) and fluid velocity (46) are as follows:

$$w_1 = 0.20403, \quad w_2 = -0.073008, \quad w_3 = -0.65676, \quad w_4 = -0.6802, \quad w_5 = 0.14507,$$

$$v_1 = 0.061258, \quad v_2 = 9.584 \times 10^{-4}, \quad v_3 = -4.939 \times 10^{-3}, \quad v_4 = -4.12 \times 10^{-3}, \tag{S6}$$

while those for the pressure function (31) remain the same as in the general case.

1.3 Toughness dominated regime with $\hat{K}_I = 10$

In the general case $0 < n < 1$ the approximation coefficients for the aperture (29), fluid velocity (30) and pressure (31) are given in the form:

$$\mathcal{Z}(n) = \frac{\sum_{k=0}^{6} r_k n^k}{\sum_{k=0}^{5} s_k n^k}, \tag{S7}$$

with the values of r_k, s_k for the case $0 < n < 1$ being listed in Tables S7 and S8.

For a Newtonian fluid $n = 1$ the coefficients for the approximate pressure function (43) are now given by:

$$p_1 = 8.86228, \quad p_2 = 9.23151 \times 10^{-6}, \quad p_3 = -1.384716 \times 10^{-5}, \quad p_4 = -8.6771 \times 10^{-11}, \tag{S8}$$

while those for the aperture (29) and fluid velocity (30) remain the same as in the general case.

Finally, in the case of a perfectly plastic fluid $n = 0$ the coefficients used to approximate the aperture (48) and fluid velocity (46) are as follows:

$$w_1 = 2.2352 \times 10^{-3}, \quad w_2 = -7.2 \times 10^{-4}, \quad w_3 = -6.893 \times 10^{-3},$$

$$w_4 = -7.137 \times 10^{-3}, \quad w_5 = 1.843 \times 10^{-3}, \tag{S9}$$

$$v_1 = 7.040647 \times 10^{-3}, \quad v_2 = 2.8 \times 10^{-7}, \quad v_3 = -8.3 \times 10^{-6}, \quad v_4 = -8.4 \times 10^{-6},$$

while those for the pressure function (31) remain the same as in the general case.

$\mathcal{Z}(n)$	r_0	r_1	r_2	r_3	r_4	r_5	r_6
w_1	0.01306626	0.0775474	-0.05721985	-0.0640863	0.082593	-0.01774346	0
w_2	0.0878826	-0.1161146	0.232977	-0.2632192	0	0	0
w_3	-0.02452076	-0.0963696	0.465517	-0.768842	0.916261	-0.76577	0.376085
w_4	1.044817×10^{-3}	-0.0625069	0.0359431	1.138043×10^{-3}	-4.5913×10^{-3}	0	0
w_5	0.1573167	-0.383264	0.240315	-0.1076469	0.1434914	-0.126323	0.03972025
v_1	-5.33568×10^{-3}	0.0490222	0.1536204	0.1200713	2.598823×10^{-3}	0	0
v_2	1.116405×10^{-3}	-1.752448×10^{-3}	9.66676×10^{-4}	-1.993698×10^{-4}	0	0	0
v_3	5.16309×10^{-3}	-3.942155×10^{-3}	2.435618×10^{-3}	0	0	0	0
v_4	8.42106×10^{-3}	-4.082466×10^{-3}	-0.0754209	0.01562056	-0.01000874	2.352954×10^{-3}	0
p_1	13.254	-13.317	0	0	0	0	0
p_2	-0.1896	0.368634	-0.17891	0	0	0	0
p_3	2.85655	-3.3178	0.96667	0	0	0	0
p_4	-1.41826	1.60526	-0.500383	0	0	0	0

Table S5: The values of coefficients $r_1, \ldots, r_6$ used in approximation (S4) in the general case $0 < n < 1$ with $\hat{K}_I = 1$.

$\mathcal{Z}(n)$	r_7	s_0	s_1	s_2	s_3	s_4	s_5
w_1	0	0	-7.11987 $\times 10^{-5}$	0.5363626	1	0	0
w_2	0	-1.036047$\times 10^{-3}$	7.22007	-2.613708	12.86834	-5.560094	1
w_3	-0.0811716	0	0	1	0	0	0
w_4	0	1	0	0	0	0	0
w_5	0	1	0	0	0	0	0
v_1	0	1.128027$\times 10^{-5}$	0.891702	1.62677	1	0	0
v_2	0	-1.066292$\times 10^{-5}$	0.865467	1.724926	1.837862	1	0
v_3	0	3.128464$\times 10^{-5}$	0.861807	0.02594735	1.430577	-0.703677	1
v_4	0	-9.85603$\times 10^{-6}$	1.79581	1	0	0	0
p_1	0	14.6064	-11.804	-7.0187	7.3799	-4.1367	1
p_2	0	5.4791	4.8663	-4.7430	-1.34197	-4.34704	1
p_3	0	6.47756	5.5563	-5.9133	-2.0187	-5.0902	1
p_4	0	4.14283	5.2029	-1.85248	-2.80587	-5.6837	1

Table S6: The values of coefficients r_7 and s_k used in approximation (S4) in the general case $0 < n < 1$ with $\hat{K}_I = 1$.

$\mathcal{Z}(n)$	r_0	r_1	r_2	r_3	r_4	r_5	r_6
w_1	2.091489×10^{-5}	-2.17337×10^{-5}	0	0	0	0	0
w_2	1.576727×10^{-5}	-2.197917×10^{-5}	0	0	0	0	0
w_3	-1.305999×10^{-5}	1.981974×10^{-5}	0	0	0	0	0
w_4	9.1626×10^{-6}	-7.9008×10^{-4}	3.71779×10^{-3}	-7.84178×10^{-3}	8.92224×10^{-3}	-5.33619×10^{-3}	1.315743×10^{-3}
w_5	1078013×10^{-4}	-8.97955×10^{-4}	1.822423×10^{-4}	0	0	0	0
v_1	-9.10529×10^{-6}	6.74877×10^{-3}	0.02089243	0.01419788	-2.20858×10^{-5}	0	0
v_2	3.9550128×10^{-8}	$3.298671\ \times10^{-8}$	$-2.0487\ \times10^{-7}$	0	0	0	0
v_3	8.61754×10^{-7}	-1.455384×10^{-6}	6.6048×10^{-7}	0	0	0	0
v_4	1.769907×10^{-4}	-1.483712×10^{-3}	-0.0455816	0	0	0	0
p_1	0.884925	3.86403	8.86889	-3.30243×10^{-3}	0	0	0
p_2	-1.323758×10^{-4}	2.79849×10^{-4}	-1.506993×10^{-4}	0	0	0	0
p_3	0.02033428	-0.0862176	0.1551598	-0.1360064	0.0477962	0	0
p_4	-0.0165688	0.0751266	-0.142603	0.1293825	-0.0461426	0	0

Table S7: The values of coefficients r_k used in approximation (S7) in the general case $0 < n < 1$ with $\hat{K}_I = 10$.

$\mathcal{Z}(n)$	s_0	s_1	s_2	s_3	s_4	s_5
w_1	4.622623×10^{-5}	-1.900085×10^{-3}	0.1066525	-0.1166793	1	0
w_2	1.722383×10^{-3}	0.06665135	1	0	0	0
w_3	6.52219×10^{-5}	-2.700796×10^{-3}	0.0875316	-0.2049092	1	0
w_4	1	0	0	0	0	0
w_5	0.0569477	0.1781103	1	0	0	0
v_1	4.40059×10^{-4}	0.950784	2.494946	1	0	0
v_2	2.3357×10^{-3}	-0.0486563	1	0	0	0
v_3	3.87371×10^{-5}	0.0666403	0.369115	0.686029	1.67268	1
v_4	0.034835	14.4869	3.31537	4.40656	-3.29718	1
p_1	0.0997469	0.436514	1	0	0	0
p_2	0.03608244	0.1856344	0.3893436	1	0	0
p_3	0.4682724	1	0	0	0	0
p_4	0.382409	1	0	0	0	0

Table S8: The values of coefficients s_k used in approximation (S7) in the general case $0 < n < 1$ with $\hat{K}_I = 10$.
